# Effects of Nicotine on the Neurophysiological and Behavioral Effects of Ketamine in Humans

**DOI:** 10.3389/fpsyt.2014.00003

**Published:** 2014-01-24

**Authors:** Daniel H. Mathalon, Kyung-Heup Ahn, Edward B. Perry, Hyun-Sang Cho, Brian J. Roach, Rebecca K. Blais, Savita Bhakta, Mohini Ranganathan, Judith M. Ford, Deepak Cyril D’Souza

**Affiliations:** ^1^Department of Psychiatry, University of California San Francisco, San Francisco, CA, USA; ^2^Mental Health Service (116D), San Francisco VA Medical Center, San Francisco, CA, USA; ^3^Department of Psychiatry, Yale University School of Medicine, New Haven, CT, USA; ^4^Schizophrenia Biological Research Center (116A), VA Connecticut Healthcare System, West Haven, CT, USA; ^5^Abraham Ribicoff Research Facilities, Connecticut Mental Health Center, New Haven, CT, USA; ^6^Department of Psychiatry, Yonsei University College of Medicine, Seoul, South Korea

**Keywords:** schizophrenia, nicotine, ketamine, *N*-methyl-d-aspartate receptor, nicotinic acetylcholine receptor, event-related potential, mismatch negativity, P300

## Abstract

**Background:** *N*-methyl-d-aspartate (NMDA) receptor hypofunction has been implicated in the pathophysiology of schizophrenia and its associated neurocognitive impairments. The high rate of cigarette smoking in schizophrenia raises questions about how nicotine modulates putative NMDA receptor hypofunction in the illness. Accordingly, we examined the modulatory effects of brain nicotinic acetylcholine receptor (nAChR) stimulation on NMDA receptor hypofunction by examining the interactive effects of nicotine, a nAChR agonist, and ketamine, a non-competitive NMDA receptor antagonist, on behavioral and neurophysiological measures in healthy human volunteers.

**Methods:** From an initial sample of 17 subjects (age range 18–55 years), 8 subjects successfully completed 4 test sessions, each separated by at least 3 days, during which they received ketamine or placebo and two injections of nicotine or placebo in a double-blind, counterbalanced manner. Schizophrenia-like effects Positive and Negative Syndrome Scale, perceptual alterations Clinician Administered Dissociative Symptoms Scale, subjective effects Visual Analog Scale and auditory event-related brain potentials (mismatch negativity, MMN; P300) were assessed during each test session.

**Results:** Consistent with existing studies, ketamine induced transient schizophrenia-like behavioral effects. P300 was reduced and delayed by ketamine regardless of whether it was elicited by a target (P3b) or novel (P3a) stimulus, while nicotine only reduced the amplitude of P3a. Nicotine did not rescue P300 from the effects of ketamine; the interactions of ketamine and nicotine were not significant. While nicotine significantly reduced MMN amplitude, ketamine did not.

**Conclusion:** Nicotine failed to modulate ketamine-induced neurophysiological and behavioral effects in this preliminary study. Interestingly, ketamine reduced P3b amplitude and nicotine reduced P3a amplitude, suggesting independent roles of NMDA receptor and nAChR in the generation of P3b and P3a, respectively.

## Introduction

Several lines of evidence support a glutamatergic hypothesis of schizophrenia involving *N*-methyl-d-aspartate (NMDA) receptor hypofunction (1–7). Studies with the NMDA receptor antagonist, ketamine, in healthy human subjects have been a cornerstone of the glutamatergic hypothesis of schizophrenia, producing clinical symptoms and cognitive impairments similar to those observed in schizophrenia (4, 5, 7–13). Nicotine has been shown to have cognitive enhancing effects in some (14–16), although not all (17, 18), studies. It has been suggested that the high rate of cigarette smoking in schizophrenia patients may reflect their efforts to use nicotine to “self-medicate” (19–21). In the present, exploratory study, we examined the interactive effects of ketamine and nicotine in healthy volunteers in order to determine whether impairments in brain function mediated by NMDA receptor hypofunction can be ameliorated by nicotine.

### Parallel effects of ketamine and schizophrenia on clinical, cognitive, and neurophysiological function

When administered at subanesthetic doses, ketamine produces an array of transient effects in healthy humans that resemble the positive and negative symptoms and cognitive deficits of schizophrenia (4, 7–13, 22–26). Ketamine has also been shown to produce some of the event-related brain potential (ERP) abnormalities observed in schizophrenia including reductions of two ERP components associated with controlled and automatic processing of deviant stimuli, P300 and mismatch negativity (MMN), as described below.

P300 is a positive voltage ERP component occurring about 300 ms following infrequent deviant stimuli interspersed among frequent “standard” stimuli, typically elicited in auditory or visual “oddball” tasks (27). P300 has been posited to reflect allocation of attentional resources (28–30), stimulus categorization (31), and contextual updating of working memory (32). Multiple brain regions have been implicated as neural generators of the P300, including the temporo-parietal junction and prefrontal cortex (33, 34). P300 comprises two subcomponents, the P3b and P3a that are differentially present depending on task conditions (27, 35–38). The P3b is primarily elicited by infrequent target stimuli, reflects top-down allocation of attention, and has a parietal scalp maximum (27, 36, 37). The P3a is primarily elicited by an infrequent non-target distractor or novel stimuli in an oddball sequence, reflects bottom-up orienting of attention, and has a fronto-central scalp maximum (27, 38–41).

P300 amplitude reduction, particularly auditory P3b, is one of the most widely replicated brain abnormalities observed in patients with schizophrenia (42, 43), although it is also reduced in a number of other psychiatric and neurological disorders (44, 45). Furthermore, several studies have shown that ketamine reduces P300 amplitude in healthy volunteers (46–51), consistent with the possibility that NMDA receptor hypofunction contributes to P300 amplitude reduction in schizophrenia. Interestingly, in the Knott et al. (47) study, the reduction of P300 amplitude by ketamine was only evident in the subgroup of non-smokers, consistent with a possible protective effect of nicotine.

Mismatch negativity is a negative ERP component elicited automatically by infrequent deviant auditory stimuli randomly interspersed among frequent “standard” stimuli (52, 53). MMN has been widely interpreted to reflect auditory sensory echoic memory because the detection of deviance requires an online representation of what has recently been “standard” in the auditory stream (52). More recently, interpretations of the MMN have emphasized its reflection of both short-term (seconds) and long-term (minutes to hours) synaptic plasticity in the service of auditory sensory/perceptual learning since the amplitude of the MMN to a deviant stimulus increases as a function of the number of repetitions of the preceding standard stimulus (54, 55). From this perspective, memory traces of the recent auditory past code predictions of future auditory events, with the MMN signaling a prediction error, when the auditory expectancy is violated by a deviant stimulus (54–56). Auditory deviance along a number of dimensions elicits MMN, including pitch, duration, intensity, and location, among others (52, 57). MMN generators have been localized to the auditory cortex and to the frontal lobes (52). MMN is considered to be largely pre-attentive (52), and it is typically elicited while subjects perform a distractor task.

Mismatch negativity has been shown to be reduced in schizophrenia, particularly the duration-deviant MMN (58–60). Moreover, MMN abnormalities, relative to P300 abnormalities, appear to be more specific to schizophrenia (61, 62). Non-competitive NMDA receptor antagonism by phencyclidine (PCP) and ketamine has been shown to reduce MMN in non-human primates (63, 64) and healthy volunteers (26, 46, 65–68), respectively. However, some studies failed to find an effect of ketamine on MMN (50) or showed the effect to depend on the type of auditory deviance or the underlying cortical source examined (65, 67). Recently, Knott and colleagues (66) reported that reduction of MMN with ketamine was only seen in people with a predisposition to experience auditory hallucinations and delusions. Moreover, this effect was blocked when these subjects were chewing nicotine gum, consistent with a possible protective effect of nicotine (66).

### Effects of nicotine on neurocognitive and neurophysiological function

Nicotine has been shown to enhance cognitive functions including attention, episodic memory, and working memory in humans in some (14–16, 69, 70), but not all (17, 18), studies. The effects of nicotine administration or smoking on neurophysiological measures related to processing of deviant stimuli have been examined in a number of studies (71, 72). In experienced tobacco users, cigarette smoking or nicotine administration has been shown to increase P300 amplitude (73–75), especially in non-smokers (47), and/or to reduce P300 latency (74, 76, 77). However, some studies have failed to show such effects (78–81) or have found effects on visual, but not auditory, P300 (77). In addition, some studies (75, 82, 83), but not others (84), have shown reduced P300 amplitudes in chronic smokers, suggesting a distinction between the effects of acute and chronic exposure to nicotine. Indeed, Knott and colleagues reported that the enhancement of P300 produced by nicotine administration was only evident in non-smokers (47).

Regarding MMN, some studies have reported that nicotine or nicotinic agonists increase MMN amplitude (85–88) or shorten MMN latency (86, 89, 90), whereas others have failed to show such amplitude increases (89–91) or latency reductions (66, 88, 91) with nicotine administration.

### Nicotine and schizophrenia

There is a high prevalence of cigarette smoking among patients with schizophrenia (92–95). This may reflect an effort by schizophrenia patients to “self-medicate” clinical symptoms and a number of neurocognitive impairments including deficits in attention and memory (19) and deficient sensory gating (96). Thus, there is significant interest in developing nicotinic acetylcholine receptor (nAChR) agonists to target the neurocognitive symptoms of schizophrenia. Indeed, an alpha7 nicotinic agonist, 3-[(2,4-dimethoxy) benzylidene] Anabaseine (DMXAB) has been shown to produce significant improvements in cognitive function and P50 sensory gating in patients with schizophrenia (97).

Several studies have examined the effects of nicotine administration on MMN in patients with schizophrenia. Regarding MMN amplitude, a study of schizophrenic smokers showed that nicotine increases the amplitude and the latency of duration-deviant MMN, but shows no effect on frequency-deviant MMN (98). An earlier study from the same group examined the effects of nicotine on a mixed sample of smoking and non-smoking schizophrenia patients with high levels of auditory hallucinations and found no effects on MMN amplitudes in response to duration, frequency, and intensity deviants, although the latency of the intensity-deviant MMN was shortened by nicotine (99). Another study of non-smoking schizophrenia patients failed to show any effect of nicotine on a frequency-deviant MMN (90).

### Nicotinic receptor modulation of glutamatergic neurotransmission

One possible mechanism by which nAChR agonists might enhance neurocognitive and neurophysiological function is facilitating glutamatergic neurotransmission via presynaptic nAChR (100) or via GABA interneurons (101, 102). Nicotine or nAChR agonists have been shown to facilitate glutamatergic transmission in rat prefrontal cortex (103, 104) and hippocampus (105). Specifically relevant to this study, nicotine attenuates or reverses memory and attentional deficits induced by the NMDA receptor antagonist MK-801 (dizocilpine) in rats (106, 107). Moreover, dizocilpine blocked nicotinic enhancement of memory consolidation in mice (108). Knott et al. (47) examined the effects of nicotine and a sub-perceptual dose of ketamine on P300 in men and women, smokers and non-smokers. In non-smokers, ketamine reduced P300, an effect that did not interact with nicotine. However, in the third assessment block, following the drug infusion, nicotine increased P300 amplitude on its own but further reduced P300 when combined with ketamine. In a subsequent study, Knott and colleagues (66) found that ketamine reduces the amplitude of MMN in healthy individuals with a high propensity toward hallucinatory experiences and/or delusional thinking, an effect that was blocked by nicotine. Similar effects were not evident in individuals with a low propensity toward these psychotic symptoms.

### The rationale and hypotheses for current study

It is difficult to isolate and study the NMDA receptor hypofunction and its consequences in schizophrenia. The ketamine paradigm in healthy subjects offers a pharmacological model for investigating nicotine’s effect on putative NMDA receptor hypofunction in schizophrenia. Specifically, this study examined the effects of nAChR activation on NMDA receptor hypofunction by investigating the interactions of ketamine, a non-competitive NMDA receptor antagonist, and nicotine, a nAChR agonist, when administered to healthy volunteers in a placebo-controlled study over four test days. The neurophysiological outcome measures, chosen based on their established sensitivity to schizophrenia, consisted of: (1) two variants of the auditory P300, the P3b elicited by target stimuli and the P3a elicited by novel distractor stimuli, and (2) the MMN elicited automatically by a duration-deviant auditory stimulus. The primary and secondary hypotheses were that nicotine would attenuate the neurophysiological abnormalities and schizophrenia-like clinical symptoms induced by ketamine, respectively.

## Materials and Methods

### Research participants

The study was approved by the Institutional Review Boards of VA Connecticut Healthcare System (West Haven, CT, USA) and Yale University School of Medicine (New Haven, CT, USA). Subjects were recruited via public advertisements and were paid for their study participation. Written informed consent was obtained from all subjects. Smokers who were not interested in quitting and lifetime non-smokers who had tried nicotine in the past were both invited to participate. Subjects were medically healthy by physical examination, history, electrocardiography, and laboratory testing. They had no history of a DSM-IV Axis-I disorder (other than nicotine dependence), major current or recent (<6 weeks) life stressors, and first-degree relative with a history of psychosis. Screening procedures included the Structured Clinical Interview for DSM-IV, Non-Patient Edition (109), selected sub-tests from the Wechsler Adult Intelligence Scale (Information, Vocabulary, Block Design, Picture Completion) to provide an estimate of general level of cognitive ability (110), and the Fagerström Test for Nicotine Dependence (111) to measure the severity of nicotine dependence in smokers. Subjects were instructed to refrain from consuming psychoactive substances from 1 week prior to testing through completion of the study. An outside informant identified by the subject was interviewed to corroborate information provided by potential subjects. Urine toxicology testing was performed at screening and on the morning of each test day to rule out recent illicit substance use. Subjects were instructed to fast overnight and abstain from smoking after 11:00 p.m. prior to arrival for each test day. They were excluded if breath carbon monoxide levels were higher than 10 ppm.

Seventeen subjects participated in at least one test day. Eight of the 17 subjects scheduled for four test days did not complete testing: 3 of 5 (60%) non-smokers and 5 of 12 (42%) smokers. The reasons for discontinuation were mostly related to adverse effects of ketamine or nicotine (*n* = 6). Adverse events and study discontinuations were reported to the VA Connecticut Human Studies Subcommittee. As with all of our prior ketamine studies, clinical follow-ups indicated that all adverse events associated with acute ketamine administration resolved spontaneously without any late appearing or persistent adverse effects. There were no significant differences in age, sex, education, smoking status, or Fagerström Nicotine Dependence scores between study completers and non-completers. Only nine subjects completed all four test days. Demographic data for these nine completers are presented in Table 1. Of the nine completers (five men, four women), one woman was excluded from the ERP analyses because she was too somnolent and impaired to perform the oddball task or the MMN distractor task during the Ketamine Alone test day. In addition, one man was dropped from the ERP MMN analysis because of technical problems running the MMN paradigm during his Nicotine Alone test day.

**Table 1:** **Demographic data for study completers (*N* = 9)**.

Variable	Number of subjects
Gender (male/female)	5/4
Smoking status (smoker/non-smoker)	7/2

	**Mean (SD)**

Age (years)	29.8 (7.9)
Education (years)	16.8 (3.0)
Fagerström nicotine dependence score	1.78 (2.28)

### Test days

Across the four test days, subjects received ketamine and nicotine in a double-blind, randomized, 2 × 2 crossover design. On each test day, subjects received ketamine (a bolus of 0.26 mg/kg over 1 min, followed by maintenance infusion at 0.65 mg/kg/h × 2 h) or placebo (normal saline). Fifteen minutes after the ketamine bolus, subjects received an intravenous infusion of nicotine (1.0 μg/kg/min × 10 min) or placebo followed by another nicotine or placebo injection 70 min later. The reason for two spaced nicotine injections was to attempt to minimize the possibility of desensitization that is known to occur with nicotine exposure. The dose of nicotine administered with each infusion (1.0 μg/kg/min × 10 min) = 0.7 mg in a 70-kg individual. A regular cigarette contains about 1.2–1.4 mg nicotine and an average of 0.88 mg of nicotine is delivered to a smoker from each cigarette. The timing of procedures is detailed in Table 2. Behavioral ratings were obtained at baseline and repeated periodically after the administration of ketamine and nicotine, but ERP data were collected only once per test day. Plasma ketamine and nicotine levels were measured after each infusion to rule out any pharmacokinetic interactions.

**Table 2:** **Study procedures**.

Time (minutes)	Procedure
−110	IV lines and EEG leads placed, PANSS, CADSS, VAS, VS, plasma cotinine level, breath carbon monoxide, urine toxicology, training for AX-CPT
−20	Ketamine or placebo bolus plus infusion
−19	VS, PANSS, CADSS, VAS
−5	VS
0	Nicotine or placebo
+3	VS
+5	AX-CPT, ERP recording for MMN, periodic VS
+35	Plasma ketamine and nicotine levels, VS, PANSS, CADSS, VAS
+58	VS
+60	Nicotine or placebo
+63	VS
+65	ERP recording for P300, periodic VS
+98	Plasma ketamine and nicotine levels
+100	VS
+110	PANSS, CADSS, VAS, VS
+180	PANSS, CADSS, VAS, VS
+240	VS, discharge

*PANSS, Positive and Negative Syndrome Scale; CADSS, Clinician Administered Dissociative Symptoms Scale; VAS, Visual Analog Scale; VS, vital signs; MMN, mismatch negativity; AX-CPT, AX-Continuous Performance Test*.

### Behavioral measures

The schizophrenia-like clinical symptoms induced by ketamine were assessed using the Positive and Negative Syndrome Scale (PANSS) (112). Perceptual alterations were assessed using the Clinician Administered Dissociative Symptoms Scale (CADSS) (113). The following subjective states were rated by participants using 100-mm Visual Analog Scales (VASs) (10): Talkative, Happy, Drowsy, Tense, Dad, Calm, Depressed, Anxious, Energetic, Fearful, Mellow, High, Angry, Mania, Irritable, Tired, Hungry, and Craving.

### Analysis of nicotine and ketamine levels

Plasma ketamine and norketamine were assayed using the identical method as described in detail elsewhere (114). Plasma nicotine concentrations were assayed using reversed-phase high-performance liquid chromatography (HPLC) based on a modification of a previously described method (115, 116). The nicotine assay involved HPLC/MS operated in the APCI/SIM mode using deuterated nicotine as an internal standard. After addition of the internal standard the plasma is deproteinized with sulfosalicylic acid and the supernatant made alkaline and extracted with heptane methylene chloride 85:15. This solvent is then dried down via vacuum centrifuge. The residue is redissolved in ethanol and an aliquot is injected into the HPLC. The HPLC column (Nova Pak C18 30 cm × 3.9 mm, 4 μm) is run in the isocratic mode using methanol acetonitrile ammonium formate (pH 4.0) 32.5:42.5:35.0 as mobile phase. The standard curve encompassing a range of 1–200 ng/ml was linear with negligible intercept. Plasma controls containing 4, 40, and 80 ng/ml nicotine in six consecutive runs demonstrated an inter-assay relative standard deviation RSD of 8.6, 7.4, and 8.3%, respectively.

### ERP measures

Electroencephalography (EEG) data were recorded using a 23-channel Physiometrix electrode cap. The cap included one ground electrode placed on the forehead (FPz), and the mean of freely placed bilateral earlobe electrodes served as the reference channel. Vertical and horizontal electro-oculograms (VEOGs and HEOGs) were recorded and used to correct EEG data for eye blink and eye-movement artifacts. Electrode impedances were maintained at <5 kΩ. The data were recorded using Neuroscan Synamps amplifiers, which were calibrated prior to each session. Data were acquired using a 0.05–100-Hz band pass filter, and the sampling rate was 1000 Hz.

P300 was elicited during an auditory oddball target detection task. Three types of stimuli were delivered through Etymotic ER-3A insert earphones: (1) standard tones: 500 Hz pure tones (rise/fall 5 ms; 50 ms duration), (2) target tones: 1000 Hz pure tones (rise/fall 5 ms; 50 ms duration), and (3) novel distractor sounds, selected from a corpus of novel sounds (average duration of 250 ms) developed by Friedman et al. (117). All auditory stimuli were presented at an identical sound pressure level (~80 dB SPL C scale). The task was presented in three blocks. Each block comprised of 150 pseudo-randomized stimuli (80% standards, 10% targets, 10% novel distractors) presented with a stimulus onset asynchrony (SOA) of 1250 ms. Subjects were instructed to press a response button with the index finger of their dominant hand each time a target tone occurred, giving equal emphasis to speed and accuracy.

For the MMN paradigm, subjects were presented with a pseudorandom sequence of standard tones (90% probability; 633 Hz; 5 ms rise/fall time; 50 ms duration) and duration-deviant tones (10% probability; 633 Hz, 5 ms rise/fall time, 100 ms duration) presented at 80 dB SPL, with a 510-ms SOA. A long duration-deviant MMN paradigm was chosen because of some evidence that it may more sensitive to the effect of schizophrenia than other types of MMN (58, 60, 61). The MMN paradigm was presented in two blocks, with each block comprising 783 standard tones and 87 deviant tones. These tones were presented binaurally through earphone inserts, while subjects performed a visual AX-Continuous Performance Task (AX-CPT) (118). Because several of the behavioral performance files from this task were irretrievably corrupted resulting in many subjects with missing behavioral performance data, the AX-CPT performance data were not analyzed in the current study. Thus, the AX-CPT essentially served as the distractor task during MMN recording.

### ERP data processing

**Figure 1:**
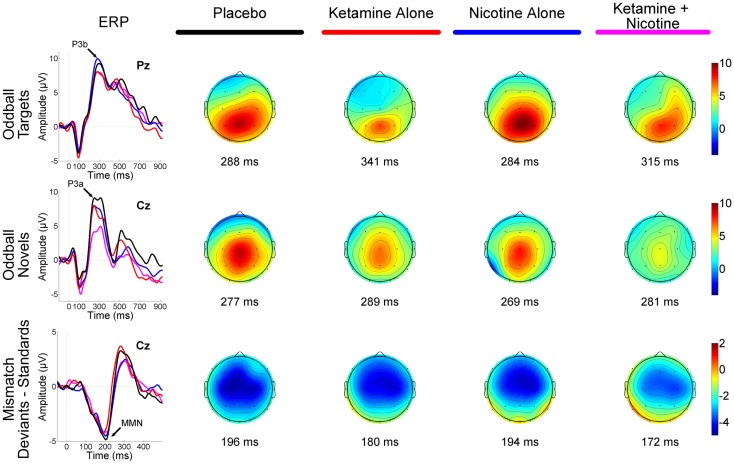
**Event-related brain potential grand average waveforms (left) and corresponding topographic maps (right) are shown for placebo (black), ketamine alone (red), nicotine alone (blue), and ketamine + nicotine (magenta) days**. ERPs, overlaid for each test day, are shown to oddball targets at Pz (top row), to oddball novels at Cz (middle row), and to difference waveforms (deviants-standards) at Cz. The oddball target elicited a P3b, the oddball novel elicited a P3a, and the deviant elicited a MMN, with each peak denoted by an arrow on the ERP waveforms. Amplitude in microvolts is on the *y*-axis, and latency in milliseconds is on the *x*-axis. Stimulus onset is at 0 ms. Negativity is plotted down. Scalp topography maps are shown for each test day for each stimulus, at the peak latency for P3b (top), P3a (middle), and MMN (bottom). Hot colors indicate positive voltage and cool colors, negative voltage.

As the MMN and P300 measures generally achieve their maximum amplitudes along the midline and do not typically show hemispheric lateralization, EEG data from the midline fronto-central sites (Fz, Cz) and fronto-central-parietal sites (Fz, Cz, Pz) were analyzed for the MMN and P300 components, respectively. The processing pipeline for the P300 elicited during the three-stimulus auditory oddball task involved the following steps: continuous data were separated into 1000 ms epochs time-locked to stimulus onset, with a 100-ms pre-stimulus baseline. VEOG and HEOG data were used to correct EEG for eye-movements and blinks with a regression-based algorithm (119). After baseline correction, epochs containing artifacts (voltages exceeding ±100 μV) were rejected. P300 was identified as the most positive peak in a 235- to 400-ms time window following stimulus onset; however, because target P3b and novelty P3a have different topographies, different rules were used for identifying their peaks. The target P3b peak was first identified at Pz, then a 50-ms window (±25 ms) surrounding this peak’s latency was used to identify target P3b peaks at other sites. Novelty P3a showed more scalp variability in peak latency than target P3b, particularly at frontal sites, leading us to adopt a more flexible peak identification approach. Novelty P3a peaks were first identified at all central and parietal sites. From the range of peak latencies obtained at central sites (C3, Cz, C4), minimum and maximum latencies were identified. By subtracting 50 ms from the minimum and adding 50 ms to the maximum, the search window for identification of P3a peaks at frontal sites was defined. Somewhat early latency cut-off (400 ms) for auditory P300s was chosen to avoid picking the second late positive component, which peaked around 550 ms (see Figure 1). Peak amplitudes and latencies for target P3b and novelty P3a were extracted from electrodes Fz, Cz, and Pz for statistical analyses.

The same eye-movement correction and artifact rejection criteria used in the P300 data processing pipeline were applied to the MMN standard and deviant trials, but these data were segmented into 550 ms epochs and baseline corrected using the 50-ms preceding tone onset. Standard and deviant trials remaining after artifact rejection were averaged separately, and the resulting ERP for the standard was subtracted from the deviant to create a difference wave. MMN was identified as the most negative peak between 90 and 270 ms post-tone onset in the difference wave at electrodes Fz and Cz. Peak amplitudes and latencies were extracted for statistical analyses.

### Statistical analyses

#### Behavioral data

Initially, behavioral data were examined descriptively using means, standard deviations, and graphs. Each measure was tested for normality using Kolmogorov-Smirnov test statistics and normal probability plots. All PANSS, CADSS, and VAS measures were highly skewed. Thus, these non-normal behavioral data were first ranked and then fitted using a mixed-effects model with an unstructured variance-covariance matrix and *p*-values adjusted for Analysis of Variance (ANOVA)-type statistics (ATS). In these models, Ketamine (active vs. placebo), Nicotine (active vs. placebo), and Time (−110, −19, +35, +110, and +180 min) were included as within-subjects explanatory factors, while Subject was the clustering factor. Time reflected the time point, in minutes, relative to Time 0 when the first intravenous infusion of active-nicotine or placebo-nicotine was initiated (see Table 2). All two- and three-way interactions were modeled. Significant interactions were followed by appropriate *post hoc* tests and graphical displays to interpret the effects. All results were considered statistically significant at *p* < 0.05. Bonferroni correction was applied within but not across domains. Thus, for example, a cut-off alpha level of 0.05/2 = 0.025 was used to declare effects significant for the two CADSS ratings (Subject- and Clinician-Rated).

#### ERP data

For the ERP data, which were collected once per test day, repeated-measures ANOVAs were conducted with Ketamine (active vs. placebo) and Nicotine (active vs. placebo) as within-subjects factors. The ANOVA models assessing P300 amplitudes and latencies included two additional within-subjects factors: Deviant Type (target vs. novel) and Lead (Fz vs. Cz vs. Pz). The ANOVA model for MMN amplitude and latency included one additional within-subjects factor: Lead (Fz vs. Cz). Analyses proceeded in a hierarchical fashion, with higher order interactions being parsed by examining lower order simple main effects and interactions. Ultimately, condition comparisons were tested with single degree of freedom contrasts.

## Results

### Behavioral data

**Figure 2:**
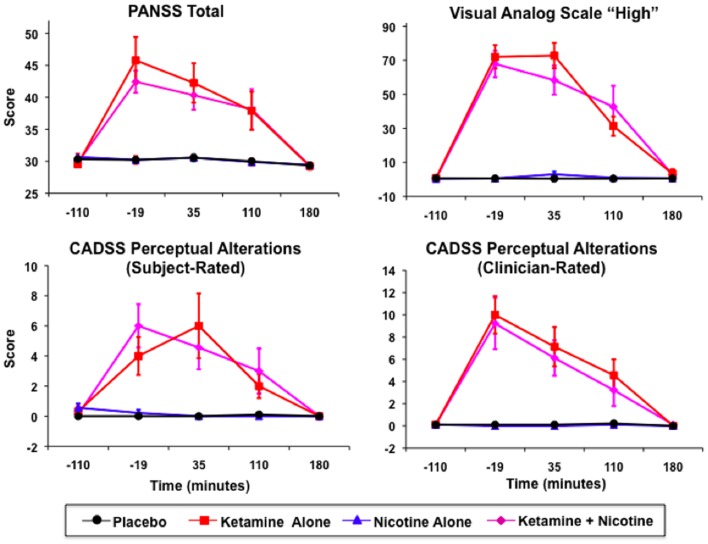
**Mean and standard errors are plotted for Positive and Negative Syndrome Scale (PANSS) total scores (upper left), Visual Analog Scale of subjective states (upper right), subject-rate (lower-left) and clinician-rated (lower-right) perceptual alterations using the Clinician Administered Dissociative Symptoms Scale (CADSS)**. For each plot, values for each of the four test days are overlaid, for Placebo (black), Ketamine Alone (red), Nicotine Alone (blue), and Ketamine + Nicotine (magenta) days.

Ketamine produced significant increases in PANSS Total, CADSS Subject-Rated and Clinician-Rated Perceptual Alterations, and VAS subjective “High” ratings (see Figure 2). All Ketamine main effects and Ketamine × Time interactions were significant at *p* < 0.0001 (Table 3). *Post hoc* analyses showed significant effects of Ketamine at time points −19, +35, and +110 (all *p* < 0.05) for each of these measures. There were no significant main effects of Nicotine. Nor were there any significant Ketamine × Nicotine or Ketamine × Nicotine × Time interactions for any of these outcome measures.

**Table 3:** **Analysis of clinical scales**.

Effect	Positive and Negative Syndrome Scale				Visual Analog Scale				CADSS perceptual alterations							
	Total score				“High”				Subject-rated				Clinician-rated			
	Num *df*	ATS	*p*-Value	Direction of effect	Num *df*	ATS	*p*-Value	Direction of effect	Num *df*	ATS	*p*-Value	Direction of effect	Num *df*	ATS	*p*-Value	Direction of effect
Nicotine (nicotine vs. placebo-nicotine)	1	0.36	0.551		1	0.59	0.441		1	0.96	0.327		1	0.20	0.655	
Ketamine (ketamine vs. placebo-ketamine)	1	24.60	**<0.0001**	Ketamine > placebo	1	77.01	**<0.0001**	Ketamine > placebo	1	23.39	**<0.0001**	Ketamine > placebo	1	43.32	**<0.0001**	Ketamine > placebo
Time (−110 vs. −19 vs. 35 vs. 110 vs. 180 min)	2.58	21.50	**<0.0001**	(−19, 35, 110) > (−110, 180)	3.39	29.94	**<0.0001**	(−19, 35, 110) > (−110, 180)	2.66	9.32	**<0.0001**	(−19, 35, 110) > (−110, 180)	2.46	19.98	**<0.0001**	(−19, 35, 110) > (−110, 180)
Nicotine × ketamine	1	0.020	0.892		1	0.190	0.660		1	0.050	0.829		1	0.100	0.751	
Nicotine × time	2.72	0.18	0.892		3.47	0.31	0.847		3.11	1.10	0.349		3.42	0.65	0.601	
Ketamine × time	3.12	22.12	**<0.0001**		3.09	22.97	**<0.0001**		3.15	16.39	**<0.0001**		2.72	23.10	**<0.0001**	
*Ketamine effect at −110 min*	*1*	*2.99*	*0.084*		*1*	*0.00*	*0.953*		*1*	*0.18*	*0.672*		*1*	*0.00*	*1.000*	
*Ketamine effect at −19 min*	*1*	*38.06*	**<*0.0001***	*Ketamine* > *placebo*	*1*	*96.57*	**<*0.0001***	*Ketamine* > *placebo*	*1*	*27.32*	**<*0.0001***	*Ketamine* > *placebo*	*1*	*142.70*	**<*0.0001***	*Ketamine* > *placebo*
*Ketamine effect at 35 min*	*1*	*41.12*	**<*0.0001***	*Ketamine* > *placebo*	*1*	*75.97*	**<*0.0001***	*Ketamine* > *placebo*	*1*	*33.83*	**<*0.0001***	*Ketamine* > *placebo*	*1*	*51.34*	**<*0.0001***	*Ketamine* > *placebo*
*Ketamine effect at 110 min*	*1*	*26.90*	**<*0.0001***	*Ketamine* > *placebo*	*1*	*29.44*	**<*0.0001***	*Ketamine* > *placebo*	*1*	*12.09*	***0.0005***	*Ketamine* > *placebo*	*1*	*15.17*	**<*0.0001***	*Ketamine* > *placebo*
*Ketamine effect at 180 min*	*1*	*0.57*	*0.449*		*1*	*3.40*	*0.065*		*1*	*0.00*	*1.000*		*1*	*0.34*	*0.559*	
Nicotine × ketamine × time	2.67	0.04	0.983		3.34	1.00	0.399		3.05	1.34	0.258		3.49	1.00	0.399	

*Results are based on a non-parametric mixed-effects model of ranked data with an unstructured variance-covariance matrix and *p*-values adjusted for ANOVA-type statistics (ATS). Follow-up test results are shown in italics. Significant p-values are shown in bold. Num *df*, numerator degrees of freedom*.

### P300 amplitude

**Figure 3:**
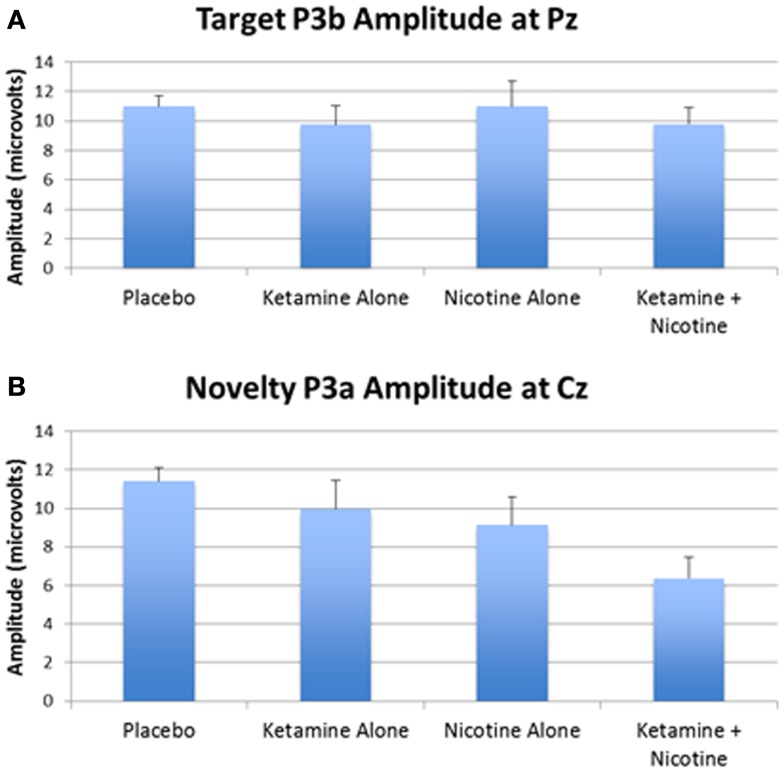
**Mean P300 amplitude across four test days**. **(A)** Mean target P3b amplitude for each test day. **(B)** Mean novelty P3a amplitude for each test day. Error bars indicate standard errors.

Event-related brain potential overlays showing P300 waves and topographic maps are shown in Figure 1, P300 peak amplitude and latency means are presented in Table 4 and histograms showing the effects of the drug conditions on P3b and P3a are shown in Figure 3. Results of the Ketamine × Nicotine × Deviant Type × Lead repeated-measures ANOVA for P300 amplitude and latency are presented in Table 5.

**Table 4:** **Means and standard errors for auditory oddball P300 amplitude and latency across the four test sessions**.

Condition	Deviant type	Lead	P300 peak			
			Amplitude (μV)		Latency (ms)	
			Mean	SE	Mean	SE
Placebo	Target P3b	Fz	4.232	0.961	283.625	12.659
		Cz	8.423	1.213	274.125	9.003
		Pz	10.981	0.713	288.500	10.544
	Novelty P3a	Fz	7.049	1.052	313.125	17.845
		Cz	11.409	0.727	277.000	12.694
		Pz	11.036	0.727	296.625	14.493
Ketamine alone	Target P3b	Fz	3.043	1.639	338.125	20.994
		Cz	5.490	2.337	333.750	21.874
		Pz	9.744	1.300	341.500	19.991
	Novelty P3a	Fz	7.356	0.935	311.125	15.725
		Cz	9.973	1.480	289.000	17.537
		Pz	8.882	0.886	311.375	13.950
Nicotine alone	Target P3b	Fz	4.698	1.120	292.375	7.426
		Cz	8.792	1.671	280.375	9.653
		Pz	10.990	1.695	284.000	7.778
	Novelty P3a	Fz	6.163	0.870	300.375	14.645
		Cz	9.142	1.471	269.125	9.875
		Pz	8.208	1.331	284.250	13.080
Ketamine + nicotine	Target P3b	Fz	4.126	0.719	311.625	21.304
		Cz	6.741	0.736	306.250	21.445
		Pz	9.790	1.119	315.000	20.376
	Novelty P3a	Fz	5.189	0.614	311.875	10.419
		Cz	6.364	1.108	280.500	13.340
		Pz	5.916	1.185	298.625	13.143

**Table 5:** **Analysis of variance (ANOVA) of P300 peak amplitudes and latencies**.

Effect	P300 peak amplitude				P300 peak latency		
	*df*	*F*	*p*-Value	Direction of effect	*F*	*p*-Value	Direction of effect
Deviant type (target vs. novel)	1.7	1.33	0.287		2.72	0.143	
Nicotine (nicotine vs. placebo-nicotine)	1.7	0.85	0.388		1.12	0.325	
Ketamine (ketamine vs. placebo-ketamine)	1.7	3.64	0.098		6.09	**0.043**	Ketamine > placebo
Lead (Fz vs. Cz vs. Pz)	2.6	61.71	**<0.001**	Fz < Cz = Pz	8.52	**0.018**	Cz < Fz = Pz
Deviant type × ketamine	1.7	0.001	0.982		2.21	0.181	
Deviant type × nicotine	1.7	5.20	0.057		0.01	0.912	
*Nicotine effect for targets*	*1.7*	*0.12*	*0.737*				
*Nicotine effect for novels*	*1.7*	*9.06*	**0.020**	*Nicotine* < *placebo*			
Deviant type × lead	2.6	26.95	**0.001**		4.59	0.062	
*Lead effect for targets*	*2.6*	*53.08*	**<0.001**	*Fz* < *Cz* < *Pz*			
*Lead effect for novels*	*1.7*	*17.69*	**0.001**	*Fz* < *Cz* = *Pz*			
Nicotine × ketamine	1.7	0.02	0.900		0.87	0.381	
Nicotine × lead	2.6	0.82	0.486		1.40	0.317	
Ketamine × lead	2.6	8.04	**0.020**		0.34	0.723	
*Ketamine effect at Fz*	*1.7*	*0.86*	*0.384*				
*Ketamine effect at Cz*	*1.7*	*5.87*	**0.046**	*Ketamine* < *placebo*			
*Ketamine effect at Pz*	*1.7*	*3.34*	*0.110*				
Deviant type × ketamine × lead	2.6	1.11	0.389		0.07	0.932	
Deviant type × nicotine × lead	2.6	2.01	0.215		0.00	1.000	
Deviant type × nicotine × ketamine	1.7	1.02	0.346		0.79	0.405	
Nicotine × ketamine × lead	2.6	0.04	0.958		0.33	0.729	
Deviant type × nicotine × ketamine × lead	2.6	3.16	0.116		0.21	0.818	

*ANOVA results are based on mutlivariate assumptions for repeated-measures, and all *F*-tests are based on Wilks’ Lambda. Follow-up ANOVA results are shown in italics. Significant p-values are shown in bold*.

In terms of main effects, only the effect of Lead was significant, with contrasts indicating equivalent P300 amplitudes at Pz and Cz that were both larger than P300 amplitude at Fz. In terms of two-way interactions, there were significant Ketamine × Lead (*p* = 0.02) and Deviant Type × Lead (*p* = 0.001) effects, with a trend (*p* = 0.057) toward a Deviant Type × Nicotine effect. The Ketamine × Lead effect was parsed by examining the main effects of Ketamine separately for each of the three midline leads, with results showing ketamine to significantly reduce P300 amplitude at electrode Cz (*p* = 0.046), but not at Fz (*p* = 0.384) or Pz (*p* = 0.11). This ketamine-induced reduction of midline vertex P300 amplitude did not significantly depend on Deviant Type (*p* = 0.389). The Deviant Type × Lead effect was parsed by examining lead effects separately for targets and novels, both of which were significant. These Lead effects reflected the typical midline scalp topographies of target P3b amplitude (i.e., Fz < Cz < Pz) and novelty P3a amplitude (i.e., Fz < Cz = Pz). The Deviant Type × Nicotine trend was parsed by examining the main effect of Nicotine for each Deviant Type separately. Nicotine significantly reduced the amplitude of novelty P3a (*p* = 0.02) but not target P3b (*p* = 0.737). No other main effects or interactions were significant.

### P300 latency

There was a significant main effect of Ketamine (*p* = 0.043) indicating that ketamine delayed P300 latency by 25.44 ms relative to placebo (Tables 4 and 5). There was also a significant main effect of Lead (*p* = 0.018) indicating that P300’s peak latency was significantly shorter at Cz than at Pz and Fz. No other main effects or interactions were significant (see Table 5).

### Mismatch negativity amplitude

**Figure 4:**
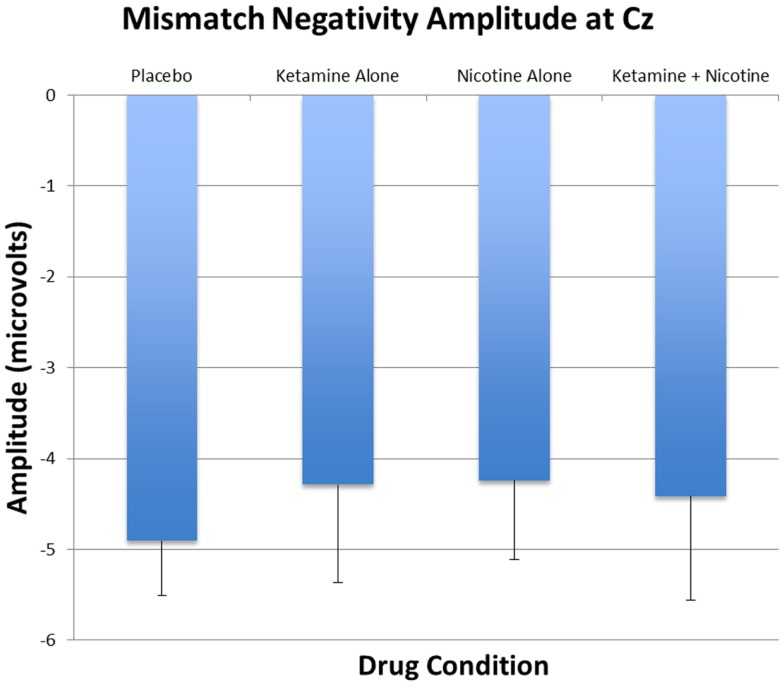
**Mean and standard errors for MMN amplitudes recorded at Cz are plotted for the four test days**.

Event-related brain potential overlays showing MMN difference waves and topographic maps are shown in Figure 1, MMN peak amplitude and latency means are presented in Table 6, and histograms showing the effects of the drug conditions on MMN are shown in Figure 4. The ANOVA results for MMN amplitude are presented in Table 7. None of the main effects were significant, but there were significant Nicotine × Ketamine and Nicotine × Ketamine × Lead interactions. The Nicotine × Ketamine × Lead three-way interaction was parsed by examining the Nicotine × Ketamine effect separately for Fz and Cz. The Nicotine × Ketamine effect was significant at Cz (*p* = 0.015) but not at Fz (*p* = 0.347). The Nicotine × Ketamine effect at Cz and the overall Nicotine × Ketamine two-way interaction (averaged over leads) were parsed by examining the main effect of each drug condition separately for the active and placebo days of the other drug condition. Nicotine Alone produced a trend level reduction of MMN amplitude relative to Placebo (*p* = 0.058 for Cz; *p* = 0.084 for average of Fz and Cz), but this Nicotine effect was not evident when Nicotine + Ketamine was compared to Ketamine Alone. In contrast, Ketamine did not significantly affect MMN amplitude when administered alone or along with Nicotine. No other interaction effects were significant.

**Table 6:** **Means and standard errors for mismatch negativity amplitude and latency across the four test sessions**.

Condition	Lead	MMN peak			
		Amplitude (μV)		Latency (ms)	
		Mean	SE	Mean	SE
Placebo	Fz	−4.591	0.687	196.286	8.216
	Cz	−4.905	0.604	196.143	7.802
Ketamine alone	Fz	−4.216	0.949	179.429	11.487
	Cz	−4.286	1.083	180.143	11.531
Nicotine alone	Fz	−4.270	0.763	201.143	7.561
	Cz	−4.239	0.873	194.143	11.754
Ketamine + nicotine	Fz	−3.890	1.012	177.571	10.040
	Cz	−4.411	1.152	172.000	11.103

**Table 7:** **Analysis of variance (ANOVA) of MMN peak amplitudes and latencies**.

Effect	*df*	MMN peak amplitude			MMN peak latency		
		*F*	*p*-Value	Direction of effect	*F*	*p*-Value	Direction of effect
Nicotine (active-nicotine vs. placebo-nicotine)	1.6	2.04	0.203		0.03	0.875	
Ketamine (active ketamine vs. placebo-ketamine)	1.6	0.20	0.668		8.77	**0.025**	Ketamine < placebo
Lead (Fz vs. Cz)	1.6	1.27	0.302		0.27	0.624	
Nicotine × ketamine	1.6	7.04	**0.038**		0.11	0.756	
*Nicotine effect during placebo-ketamine*	*1.6*	*4.27*	*0.084*	*Nicotine* < *placebo*			
*Nicotine effect during active ketamine*	*1.6*	*0.26*	*0.629*				
*Ketamine effect during placebo-nicotine*	*1.6*	*1.28*	*0.301*				
*Ketamine effect during active-nicotine*	*1.6*	*0.47*	*0.519*				
Nicotine × lead	1.6	0.01	0.914		1.81	0.227	
Ketamine × lead	1.6	0.26	0.627		0.04	0.854	
Nicotine × ketamine × lead	1.6	9.50	**0.022**		0.00	0.972	
*Nicotine* × *ketamine at Fz*	*1.6*	*1.04*	*0.347*				
*Nicotine* × *ketamine at Cz*	*1.6*	*11.38*	***0.015***				
*Nicotine effect during placebo-ketamine*	*1.6*	*5.48*	*0.058*	*Nicotine* < *placebo*			
*Nicotine effect during active ketamine*	*1.6*	*0.003*	*0.960*	*Nicotine ≥ placebo*			
*Ketamine effect during placebo-nicotine*	*1.6*	*1.40*	*0.281*	*Ketamine ≤ placebo*			
*Ketamine effect during active-nicotine*	*1.6*	*2.94*	*0.137*	*Ketamine ≥ placebo*			

*ANOVA results are based on mutlivariate assumptions for repeated-measures, and all *F*-tests are based on Wilks’ Lambda. Follow-up ANOVA results are shown in italics. Significant p-values are shown in bold*.

### Mismatch negativity latency

Analysis of variance results for MMN latency are presented in Table 7. There was a significant main effect of ketamine (*p* = 0.025) indicating that ketamine shortened MMN latency by 19.64 ms relative to placebo. No other main effects or interactions were significant.

### Plasma drug levels

Mean plasma levels for ketamine, norketamine, dehydroketamine, and nicotine are presented in Table 8. There were no significant differences in levels of plasma ketamine, norketamine, or dehydroketamine levels between the ketamine alone condition and the ketamine–nicotine condition. In addition, there were no significant differences in plasma nicotine levels between the nicotine alone condition and the ketamine–nicotine condition.

**Table 8:** **Plasma ketamine, norketamine, dehydroketamine and nicotine levels**.

Blood level (ng/ml)	Time	Placebo	Ketamine alone	Nicotine alone	Ketamine + nicotine
Ketamine	+35		174.91 (59.92)		171.22 (62.21)
	+98		222.40 (57.55)		229.78 (64.52)
Norketamine	+35		70.82 (32.61)		73.78 (40.92)
	+98		147.20 (52.21)		138.67 (45.92)
Dehydroketamine	+35		16.09 (9.86)		17.78 (9.04)
	+98		42.20 (19.77)		42.33 (23.52)
Nicotine	+35			6.92 (3.23)	10.18 (4.31)
	+98			6.65 (2.63)	12.93 (7.01)

*Ketamine levels were assayed only on the days that subjects received ketamine (ketamine alone and ketamine + nicotine) and nicotine was assayed only on the days that subjects received nicotine (nicotine alone and nicotine + ketamine)*.

## Discussion

The principal findings of the current study are the differential effects of ketamine and nicotine on MMN and P300, and their interactive effects on MMN.

### Effects of ketamine

Consistent with previous studies (4, 5, 7–13, 120), ketamine induced transient schizophrenia-like behavioral effects in healthy subjects. In terms of the electrophysiological measures, ketamine decreased the amplitude and delayed the latency of P300, regardless of whether P300 was elicited by a target or novel stimulus. The decrease in amplitude is consistent with the other ERP studies showing ketamine to reduce P300 amplitude at parietal leads (46–51), although we did not observe the previously reported increase in novelty P3a at frontal leads with ketamine (51). Our results are also consistent with a prior study showing ketamine to decrease the amplitude of the late positive potential in a working memory task (9). These results provide evidence that glutamatergic neurotransmission at NMDA receptors contribute to P300 generation, both in response to infrequent target stimuli (P3b) and infrequent novel stimuli (P3a). Moreover, inasmuch as P300 amplitude reduction and latency delay are well established in schizophrenia (42, 43, 121, 122), our findings are consistent with the NMDA receptor hypofunction model of schizophrenia (1–3, 5, 6, 123, 124) and its possible role in mediating P300 deficits.

The current study did not find ketamine to significantly reduce MMN amplitude. This conflicts with a number of previous studies (26, 46, 65–68), but is consistent with some studies that either failed to show a ketamine effect on MMN (50) or showed the ketamine-induced MMN reduction to be limited to a subset of task conditions or cortical source locations (26, 65, 67), or to a subgroup of subjects (66). The discrepant results across these studies may be due to differences in the dosage and dosing schedule of ketamine. For example, the study with the most robust effects (26) used a high dose of ketamine (0.9 mg/kg), while the study with a non-significant result (50) used a relatively low dose (0.3 mg/kg). Heekeren and colleagues (65) also demonstrated dose-dependent changes in MMN amplitude using two different doses of ketamine (0.1–0.15 and 0.15–0.20 mg/kg). The absence of a significant ketamine effect on MMN in our study may also have been related to the relatively small size of our subject sample, resulting in limited power to detect an effect. For the ketamine vs. placebo effect on MMN amplitude during the placebo-nicotine day, the effect size (Cohen’s *d*) was estimated to be −0.43. This effect size, which appears to be smaller than the effects reported in prior studies showing ketamine to reduce MMN, would reach significance (*p* < 0.05) with a sample of about 25 subjects. This underscores the limited power in our current study, and points to an effect of ketamine on MMN that may emerge with moderate sample sizes.

However, it is noteworthy that our sample size was sufficiently large to detect robust psychotomimetic effects of ketamine, as well as significant ketamine-induced reductions and delays in the P300 ERP component. Thus, consistent with the report of Oranje and colleagues (50), the P300 ERP component appears to be more sensitive to the effects of NMDA receptor blockade than the MMN component. In terms of MMN latency, ketamine reduced MMN latency by about 19 ms. This effect has not been previously reported to our knowledge, and therefore should be regarded as preliminary pending replication in future studies.

### Effects of nicotine

To our knowledge, this is the first study to show nicotine to reduce the novelty P300 (P3a) in humans. This unexpected finding appears to conflict with the plethora of evidence showing nicotine to enhance cognitive functions, including attention (14–16). It is possible to construe the novelty P3a reduction by nicotine as a reflection of enhanced focus on the target detection task and reduced susceptibility to distraction by non-target distractors. However, such an interpretation is not consistent with other evidence showing P3a reduction in patients with schizophrenia (43, 121, 125–128) and patients with frontal lobe lesions (129–131), two conditions known to be associated with attentional impairments and increased distractibility. Thus, nicotine’s reduction of the P3a response to novel distractors is unlikely to be a reflection of its cognitive enhancing effects.

We did not observe significant effects of nicotine on target P300 (P3b), inconsistent with some prior reports showing nicotine to increase P300 amplitude (47, 73, 74) and decrease P300 latency (75, 77) in smokers. However, our results are consistent with other studies reporting no effects of nicotine on P300 (76, 78–80). Our study was relatively unique in its use of the intravenous route for nicotine administration, which may partially account for inconsistencies between our results and some prior studies. More generally, inconsistencies among studies may also be related to differences in the type of nicotinic agonist and dosage used, differences in sensory modality of the oddball task used to elicit the P300, and different representations of smokers and non-smokers in the study samples.

The differential effects of nicotine on P3a and P3b in our study is consistent with other evidence that the neuroanatomical (33) and neurochemical (71) underpinnings of P3a and P3b are at least partially dissociable. Polich and Criado (71) proposed dopaminergic/frontal processes for P3a generation and locus coeruleus-mediated noradrenergic/parietal processes for P3b generation. Evidence for this includes demonstrations that chronic abuse of different street drugs are associated with differential effects on P3a and P3b amplitudes (71). Nonetheless, roles for nicotinic cholinergic neurotransmission, as well as glutamatergic neurotransmission, have not figured prominently in prior neurochemical models of P300 generation.

Nicotine alone produced a trend level reduction of MMN amplitude, but this effect was not evident when comparing the Nicotine + Ketamine condition to Ketamine alone. These results conflict with some prior studies showing nicotine or nicotinic agonists to enhance MMN amplitude in response to duration (85), frequency (86, 87), or inter-stimulus interval (88) deviants in healthy volunteers, and similarly failed to corroborate studies showing nicotine to enhance duration-deviant MMN amplitude in schizophrenia patients (98). One possible reason for the discrepancy between our findings and those reported previously is that our study used an intravenous route of nicotine administration whereas prior studies used either gum (85, 88, 98) or a transdermal patch (87). While differences in the pharmacokinetics and pharmacodynamics between intravenous vs. gum and transdermal routes of administration have not been systematically studied’, it is likely that time to onset of action and peak levels achieved would differ between these modes of nicotine delivery, and such differences could account for variability in the effects of nicotine on MMN. At the same time, it should be noted that a number of studies using gum (47, 99) or transdermal patches (89, 90) failed to demonstrate an enhancement of MMN amplitude by nicotine. Moreover, the fact that our study focused on duration-deviant MMN, in part because of evidence that it is more sensitive to schizophrenia than other types of MMN (58, 60, 61) cannot be the reason we failed to observe enhancement by nicotine, since at least two prior studies have shown the amplitude of the duration-deviant MMN to be increased by nicotine [Ref. (85, 98); but, see Ref. (99)].

### Combined effect of ketamine and nicotine

Discordant with the study hypothesis, nicotine did not improve either the behavioral or neurophysiological abnormalities induced by ketamine. Of the many drugs tested in the ketamine model, few have been shown to reduce the schizophrenia-like behavioral and cognitive effects of ketamine in healthy human subjects. Lamotrigine, but not haloperidol or lorazepam, has been shown to reduce some of the behavioral and cognitive symptoms induced by ketamine in healthy volunteers (132–134). With previous findings from animal and human studies documenting cognitive enhancing effects of nicotine in humans (135–138) and animals (139, 140), including animal data showing nicotine to ameliorate NMDA-antagonist induced cognitive deficits (106, 107) or NMDA-antagonists to block cognitive enhancing effects of nicotine (108), it was surprising that nicotine did not show any tendency to reverse ketamine’s psychotomimetic or cognitive ERP effects. Inconsistencies among studies may be due to differences in nicotine dose, rate, and route of nicotine administration, and the smoking status of the subjects tested. Importantly, our results are consistent with other studies showing that nicotine did not block ketamine’s deleterious effects on P300 (47) or on neurocognitive test performance (141) in humans, suggesting that any pro-cognitive effects of nicotine may not be able to overcome the impairments produced by NMDA receptor blockade. However, our results were not consistent with a prior study that showed nicotine to prevent ketamine’s reduction on MMN amplitude, an effect that was only observed in the subgroup of healthy volunteers with a high propensity to have hallucinatory experiences (66). However, this prior study used a substantially lower dose of ketamine than used in the current study, and nicotine was administered with chewing gum rather than the intravenous route employed here.

### Limitations

The main limitations of the current study include the small sample size, the high dropout rate, the heterogeneous smoking status of our sample, and the use of only one dose of nicotine. Future studies aimed at elucidating the effects of nicotine on patients with schizophrenia by using pharmacological models of psychosis in healthy volunteers must consider the fact that the large majority of schizophrenia patients are significantly dependent on nicotine. Accordingly, for studies about nAChR function to be relevant to schizophrenia, heavy smokers need to be included in the subject sample. However, the inclusion of nicotine-dependent heavy smokers in such studies raises the question of when to schedule the nicotine challenge relative to the timing of their last cigarette. Studying smokers who have been asked to abstain from smoking for several hours or more prior to study onset would mean studying them in a nicotine-withdrawal state. On the other hand, studying smokers who have smoked recently and are in a nicotine-satiated state may obscure the effects of intravenous nicotine. Further complicating this issue, studying non-smokers would result in high dropout routes because nicotine is generally unpleasant to non-smokers. Moreover, data from non-smokers may not generalize to schizophrenia patients, most of whom are heavy smokers.

In conclusion, the results of this study suggest that activation of nACH receptors does not influence ketamine’s psychotomimetic effects or physiological effects on MMN and P300 in healthy human volunteers. However, ketamine and nicotine appear to have independent effects on P3a, P3b, and MMN suggesting differential effects of nACH and NMDA receptor systems on these ERP components. Moreover, this is the first study to report a significant reduction in P3a amplitude by nicotine.

## Conflict of Interest Statement

The authors declare that the research was conducted in the absence of any commercial or financial relationships that could be construed as a potential conflict of interest.
